# Postoperative intestinal intussusception in children, an easily missed culprit of postoperative intestinal obstruction: Case series and literature review

**DOI:** 10.1016/j.ijscr.2019.06.057

**Published:** 2019-06-28

**Authors:** Sadi A. Abukhalaf, Tareq Z. Alzughayyar, Muath A. Baniowda, Radwan Abukarsh, Ihsan Ghazzawi, Nathan M. Novotny, Ahmad Al Hammouri

**Affiliations:** a Al-Quds University, Faculty of Medicine, Jerusalem, Palestine; b Palestine Red Crescent Society Hospital, Hebron, Palestine; c Section of Pediatric Surgery, Beaumont Children’s, Oakland University William Beaumont School of Medicine, Royal Oak, MI, USA; d Palestine Medical Complex, Ramallah, Palestine

**Keywords:** Postoperative intestinal intussusception, Postoperative intestinal obstruction, Ileocolic, Ileoileal, POI

## Abstract

**Background:**

Postoperative intestinal intussusception (POI) is a rare cause of intestinal obstruction with POI after surgical reduction of ileocolic intussusception being an extremely rare variant. POI was reported to follow many abdominal and non-abdominal operations. A late diagnosis can risk ischemia and necrosis. POI also increases the morbidity and mortality, rendering an early diagnosis and prompt management as lifesaving.

**Methods:**

We reviewed the medical charts retrospectively for the last ten years for patients with POI at Palestine Red Crescent Society Hospital, Hebron, Palestine. We reviewed the literature and presented the characteristics of the most reported cases of POI following surgical reduction of ileocolic intussusception.

**Results:**

We presented three cases of ileoileal POI and one case of ileocolic POI followed different primary operations. All but one patient presented in the first two weeks. The delayed presentation came two months after revision of a prolapsed colostomy. All patients managed successfully with operative manual reduction with no postoperative complications. Initially, we had struggles in the diagnosis of POI largely due to a low suspicion for this rare entity, but thereafter we kept POI in mind and managed the after-coming cases in an expeditious manner.

**Conclusion:**

Frequently, POI is misdiagnosed as postoperative adhesive obstruction. POI is challenging in diagnosis and needs a very high index of suspicion, mainly due to its rarity and atypical presentation. By keeping the possibility of POI in mind, one can easily diagnose it and prevent its consequences.

## Highlights

• Most of our patients presented in the first two weeks after the primary operation and managed successfully with operative manual reduction with no postoperative complications.
• Postoperative intestinal intussusception secondary to surgical reduction of ileocolic intussusception is an exceedingly rare cause of postoperative intestinal obstruction.
• Usually, postoperative intestinal intussusception is misdiagnosed as postoperative adhesive obstruction.
• Postoperative intestinal intussusception is challenging in diagnosis and needs a very high index of suspicion.
• By keeping the possibility of POI in mind, one can easily diagnose it and prevent its consequences.

## 1 Introduction

There are many etiologies for intestinal intussusception though it is largely idiopathic in origin [1]. Less common causes include POI with reported incidence after laparotomies of 0.01 to 0.25% [2]. POI presents with bilious vomiting, high nasogastric tube output, abdominal pain or abdominal distension during the first two weeks postoperatively in 90% of patients [3,4]. Because POI is a forgotten cause of postoperative obstruction, and to increase the awareness of this rare entity, we present three cases of ileoileal POI and one case of ileocolic POI.

## 2 Case presentation

### 2.1 Case 1 [POI following a colostomy]

A two year-old female patient underwent exploratory laparotomy with end sigmoid colostomy after a penetrating rectal injury. The intraoperative and early postoperative periods were uneventful. After ten days following the surgery, the child presented three times with a picture of intestinal obstruction. However, at each time, she was admitted for two days, treated conservatively, and improved. One month following the surgery, she developed diffuse severe colicky abdominal pain with vomiting, diarrhea and abdominal distension. Abdominal standing x-ray showed multiple air fluid levels with dilated loops. She was taken to the operating room and underwent a laparotomy and found an ileoileal intussusception. Manual reduction and resection of the necrotic part were performed. The child’s postoperative course was uneventful.

### 2.2 Case 2 [POI following ileocolic intussusception]

**Fig. 1:**
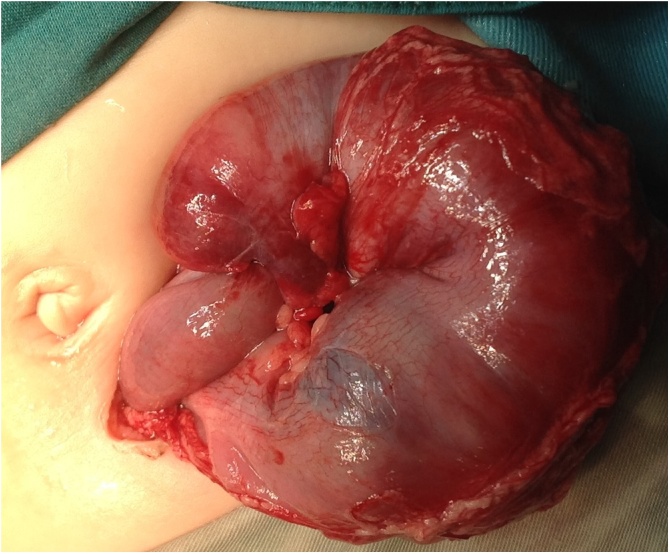
Intraoperative photograph showing ileocolic intussusception.

**Fig. 2:**
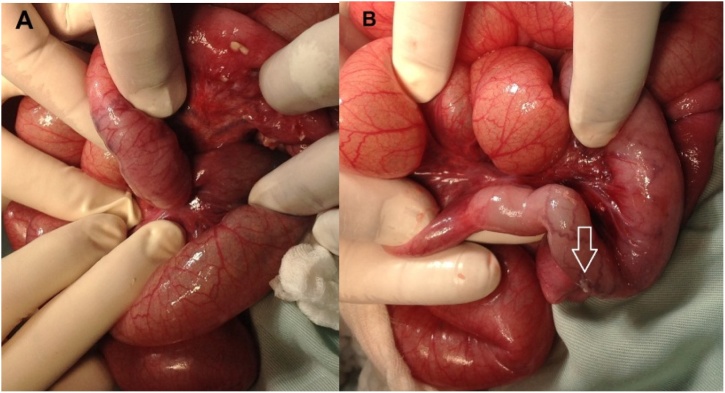
Intraoperative photographs showing ileoileal intussusception with perforation and necrosis [arrowhead].

A five-month-old female underwent surgical reduction of idiopathic ileocolic intussusception (Fig. 1). Initially, the infant did not tolerate the slow advancement of her diet and this was managed as postoperative ileus. The infant developed bilious vomiting on postoperative day seven. Her abdominal x-ray showed multiple dilated bowel loops. Abdominal ultrasound showed ileoileal intussusception. At laparotomy, an ileoileal intussusception was identified and reduced manually, with resection of a short necrotic segment (Fig. 2). Subsequently, the infant did very well and was discharged home.

### 2.3 Case 3 [POI following revision of an ileostomy]

A seven month-old male with Hirschsprung’s disease, underwent a loop ileostomy at the age of 12 days due to intestinal perforation. At the age of three months, the infant presented with prolapse of his ileostomy necessitating revision of the ileostomy. Two months following laparotomy, the infant developed a prolonged course of watery diarrhea and malabsorption with poor weight gain. Cow's milk protein allergy was suspected and formula was changed with no improvement. At the age of seven months, the infant underwent pull through procedure. An ileocolic intussusception was identified incidentally and managed by manual reduction. The infant’s stool production and intestinal absorption normalized after the reduction. His postoperative course was uneventful.

### 2.4 Case 4 [POI following a colostomy]

A six month-old male with Hirschsprung’s disease, underwent laparotomy with leveling colostomy. Three days after the operation, the infant developed abdominal distention with bilious vomiting. Plain abdominal x-ray was performed and showed multiple air fluid levels with dilated bowel loops. The infant failed to improve with conservative therapy. The patient was taken back to the operating room five days after the initial laparotomy and an ileoileal intussusception was identified and reduced manually. The infant progressed well postoperatively and was discharged home at POD5.

Table 1, Table 2 summarize the clinical details and symptoms and signs experienced by our patients. Unfortunately, we did not perform genetic testing for any patient.

**Table 1:** Patient Demographics.

Case no.	Case 1	Case 2	Case 3	Case 4
Gender (M/F)	F	F	M	M
Age (months)	25	5	7	6
Initial diagnosis	Penetrating rectal injury	Ileocolic intussusception	Loop ileostomy prolapse	Hirschsprung’s disease
Initial procedure	Laparotomy with end sigmoid colostomy	Reduction with right hemicolectomy	Laparotomy with revision of prolapsed ileostomy	Laparotomy, creation of end colostomy
Onset of symptoms	POD 10	POD 5	2 months post operation	POD 3
Day of reoperation	One month following initial procedure	One week following initial procedure	Four months following initial procedure	Five days following initial procedure
Type of intussusception	Ileoileal	Ileoileal	Ileocolic	Ileoileal
Complications of intussusception	Patches of necrosis	Perforation with 3 areas of patchy necrosis	None	None
Repair	Manual reduction with resection of necrotic areas part	Manual reduction with primary repair of perforation	Manual reduction	Manual reduction

**Table 2:** Symptoms and signs experienced by the studied patients.

	Case 1	Case 2	Case 3	Case 4
Abdominal pain	+	+	+	+
Abdominal distention	+	+	+	+
Diarrhea and mal-absorption	----	----	+	----
Palpable mass	----	----	----	----
Poor weight gain	----	----	+	----
Rectal bleeding	----	----	----	----
Vomiting	+	+	+	+

## 3 Discussion

Postoperative intestinal obstruction is a common problem encountered in children and is mostly attributed to intestinal adhesions and adynamic ileus [5,6]. One forgotten cause of postoperative intestinal obstruction is postoperative intestinal intussusception [7]. Postoperative intestinal intussusception (POI) was reported to follow many abdominal and non-abdominal operations. 51.2% and 20.5% of POI occurs in gastrointestinal tract and retroperitoneal tumor resection procedures respectively. Laparotomy, surgical reduction of ileocolic intussusception, and Hirschsprung’s disease repair operations were all previously reported to be associated with POI [2].

The reported incidence of POI is 0.01–0.25% [2] with higher incidence rates in pancreatic resection operations of 2.1% [8] and abdominal tumor resection operations of 1.2% [4]. POI has higher incidence rates in males [2] and mentally disabled patients [6]. POI is not only a complication of pediatric procedures, it also has been reported in adult procedures albeit less commonly [9].

Being reported after many different primary surgeries, POI can have different underlying mechanisms. Although etiology of POI remains unclear, several theories were proposed to explain its pathophysiology, including early postoperative adhesions, excessive bowel manipulation, altered peristalsis, neurogenic factors, electrolyte disturbances, and medications (anesthetics and opioids) [10]. The most common site of POI is the small bowel with ileoileal intussusception predominance [2]. Other reported POI sites are jejunojejunal, jejunoileal, ileocolic and multiple

intussusceptions. Ileoileal POI is frequently reported with abdominal procedures while ileocolic POI being reported more commonly with non-abdominal procedures [3].

The typical presentation of the idiopathic intussusception involves painful abdominal cramps, vomiting, a palpable abdominal mass, and rectal bleeding [2]. Lethargy and altered level of consciousness are reported as well [11].

Table 3 shows characteristics of non-postoperative intussusception and postoperative intussusception.

**Table 3:** Characteristics of non-postoperative intussusception and postoperative intussusception.

	Non-postoperative Intussusception	Postoperative Intussusception
Causes/Risk Factors	Largely idiopathic; Identified lead points	Excessive bowel manipulation, Altered peristalsis, Electrolyte disturbances, and Medications; No identified lead points
Symptoms/Signs	Triad of pain, palpable abdominal mass, and currant-jelly stool; Vomiting, Lethargy, and Altered level of consciousness	‘Prolonged adynamic ileus’, Bilious vomiting, Abdominal distension, Increased bilious nasogastric tube output, Restlessness, Bloody stools and Palpable abdominal mass
Diagnostic Tools	Abdominal ultrasonography, Abdominal radiograph, and CT scan	Requires a high index of suspicion; Contrast study, Abdominal ultrasonography, CT scan and Abdominal radiograph
Management	Non-operative reduction, Manual reduction; Bowel resection if needed	Manual reduction; Non-operative reduction is not indicated except for ileocolic POI following non-abdominal operations; Bowel resection if needed
Outcomes	Satisfactory if managed promptly	Satisfactory if managed promptly
Recurrence Rate	10–15% with non-operative and operative reduction	Unclear, but very low [10]

Unlike idiopathic intussusception, the POI frequently presents with non-specific “prolonged adynamic ileus’’ [12] symptoms with bilious vomiting being the most commonly reported presentation. Abdominal distension and increased bilious nasogastric tube output are other common presentations, with rare reporting of restlessness, bloody stools and palpable abdominal mass [2,6]. Frequently, there is no identifiable lead point [13].

POI is challenging in diagnosis and needs a high index of suspicion, mainly due to its rarity, atypical presentation, and the abundance of postoperative adynamic ileus [6,13,14]. Frequently, POI is misdiagnosed as postoperative adhesive obstruction [5,15]. Helpful diagnostic tools may include abdominal radiograph, abdominal ultrasonography, contrast study and computerized tomography (CT) scan [3]. Abdominal radiographs may demonstrate air-fluid levels although it is of little diagnostic yield [3,5,16]. One can use abdominal ultrasonography to differentiate a mechanical obstruction from other causes of obstruction (i.e. ileus) with a high specificity of 100% and sensitivity of 89% [4,6]. Contrast studies are diagnostic in up to 95% of cases of small-bowel intussusception [12]. However, in one of our patients, the contrast study was not diagnostic.

Although 90% of POI patients present within the first two weeks following the operation [4], one of our patients had ileocolic POI two months after prolapsed ileostomy revision. One study reported a similar case with three months duration postoperatively [6]. However, some authors may consider this as a coincidental idiopathic intussusception occurring during postoperative period [3].

Table 4 shows characteristics of the previously reported ileoileal POI secondary to surgical reduction of ileocolic intussusception. Seven of nine patients were male with mean age of 6.2 months. All patients presented initially with bilious vomiting and abdominal distention. All patients presented and were managed successfully with manual reduction within the first week following the primary procedures. Interestingly, all cases of POI were reported to follow surgical reduction of ileocolic intussusception. But, no cases were reported to follow surgical reduction of ileoileal or other types of intestinal intussusception.

**Table 4:** Characteristics of the previously reported ileoileal POI secondary to surgical reduction of ileocolic intussusception.

	Gender	Age	Onset of symptoms	Day of reoperation	Signs and symptoms	Second operation
Case 1 [14]	M	6 mon	POD 3	POD 5	BV, PAD and NFD	Manual reduction
Case 2 [14]	M	3 mon	POD 4	POD 8	BV, PAD and NFD	Manual reduction
Case 3 [14]	M	10 mon	POD 4	POD 6	BV, PAD and NFD	Manual reduction
Case 4 [14]	M	7 mon	POD 4	POD 6	BV, PAD and NFD	Manual reduction
Case 5 [14]	M	10 mon	POD 2	POD 3	BV, PAD and NFD	Manual reduction, appendectomy
Case 6 [14]	F	5 mon	POD 3	POD 6	BV, PAD and NFD	Manual reduction, wound secondary suture
Case 7 [5]	M	5 mon	POD 2-9	Unknown	BV, PAD, NFD and INGTD	Manual reduction with possible bowel resection
Case 8 [5]	M	5 mon	POD 2-9	Unknown	BV, PAD, NFD and INGTD	Manual reduction with possible bowel resection
Case 9 [this study]	F	5 mon	POD 5	POD 7	BV, PAD	Manual reduction

BV[Bilious vomiting], PAD[progressive abdominal distention], NFD[no fecal discharge], INGTD[increased nasogastric tube drainage].

A late diagnosis of POI poses a risk of ischemia and necrosis, and need for subsequent bowel resection. It also increases the morbidity and mortality [2,5,17], underscoring the need for early diagnosis and prompt management [14]. The mortality of POI was found as high as 6%–7% [15].

POI is usually managed by operative manual reduction with resection and anastomosis in select cases [2,10]. Two of our patients were found to have patches of necrosis and one of them needed a bowel resection. Interestingly, some reported cases resolved spontaneously [3,18]. Hydrostatic reduction can be employed in cases of non-abdominal operations or abdominal operations without anastomoses [2,6]. Suggested preventative measures for POI include gentle handling, avoidance of desiccation of the bowel and using a minimally invasive approach [3,10].

## 4 Conclusion

Frequently, POI is misdiagnosed as postoperative adhesive obstruction. POI is a rare cause of intestinal obstruction with POI after surgical reduction of ileocolic intussusception being an extremely rare variant. POI is challenging in diagnosis and needs a very high index of suspicion. Contrast studies and abdominal ultrasonography are diagnostic in the majority of cases.

## Conflicts of interest

The following authors have no financial disclosures: Sadi A. Abukhalaf, Tareq Z. Alzughayyar, Muath A. Baniowda, Radwan Abukarsh, Ihsan Ghazzawi, Nathan M. Novotny and Ahmad Al Hammouri.

## Funding

No funding or grant support.

## Ethical approval

The study is exempt from ethnical approval in our institution.

## Consent

The patient consents were all obtained by the infants’ parents. And all parents accepted the final edition of the article.

## Author contribution

Study concept or design: Radwan Abukarsh, Ihsan Ghazzawi.

Data collection and data analysis: Sadi A. Abukhalaf, Tareq Z. Alzughayyar, Muath A. Baniowda.

Writing the paper: Sadi A. Abukhalaf, Ahmad Al Hammouri, Nathan M. Novotny.

## Registration of research studies

We registered the study at http://www.researchregistry.com. with registration number of researchregistry4965 and the primary investigator is Sadi Abukhalaf. Here is the link : https://www.researchregistry.com/register-now#home/registrationdetails/5d0c0452c404ef000afa1e4b/

## Guarantor

Dr. Sadi A. Abukhalaf.

## Provenance and peer review

Not commissioned, externally peer-reviewed.
